# SNP Formation Bias in the Murine Genome Provides Evidence for Parallel Evolution

**DOI:** 10.1093/gbe/evv150

**Published:** 2015-08-06

**Authors:** Zackery E. Plyler, Aubrey E. Hill, Christopher W. McAtee, Xiangqin Cui, Leah A. Moseley, Eric J. Sorscher

**Affiliations:** ^1^Department of Biology, University of Alabama at Birmingham; ^2^Department of Computer and Information Sciences, University of Alabama at Birmingham; ^3^Gregory Fleming James Cystic Fibrosis Research Center, University of Alabama at Birmingham; ^4^Department of Biostatistics, University of Alabama at Birmingham; ^5^Department of Pediatrics, Emory University School of Medicine

**Keywords:** parallel evolution, mutation, SNP formation bias

## Abstract

In this study, we show novel DNA motifs that promote single nucleotide polymorphism (SNP) formation and are conserved among exons, introns, and intergenic DNA from mice (Sanger Mouse Genomes Project), human genes (1000 Genomes), and tumor-specific somatic mutations (data from TCGA). We further characterize SNPs likely to be very recent in origin (i.e., formed in otherwise congenic mice) and show enrichment for both synonymous and parallel DNA variants occurring under circumstances not attributable to purifying selection. The findings provide insight regarding SNP contextual bias and eukaryotic codon usage as strategies that favor long-term exonic stability. The study also furnishes new information concerning rates of murine genomic evolution and features of DNA mutagenesis (at the time of SNP formation) that should be viewed as “adaptive.”

## Introduction

Mutations and specific constraints that govern sites of DNA polymorphism are not well understood. Although eukaryotic single nucleotide polymorphism (SNP) formation is viewed as stochastic, preferences in the nature and location of certain single base replacements (including C⇔T and A⇔G [transition] mutations) are described among metazoans (Lederberg JA and Lederberg EM 1952; Collins and Jukes 1994; Wakeley 1994; Drake et al. 1998; Freeland and Hurst 1998; Sung et al. 2012; Hill et al. 2014). Isolated “hot spots” for single nucleotide variants exist, but there is little or no information regarding the overall contribution of such regions to eukaryotic SNP formation. A bias underlying hominid or murine single base polymorphism as determined by neighboring sequence context has been suggested, including effects from nucleotides upwards of 200 bp away from a polymorphic site (Koch 1971; Krawczak et al. 1998; Zhao and Boerwinkle 2002; Zhao and Zhan 2004; Zhang and Zhao 2005). Others have concluded that although human and nonhuman primate SNPs exhibit striking homology to each other, the surrounding DNA context is not responsible (Duret 2009; Hodgkinson et al. 2009; Hodgkinson and Eyre-Walker 2010). These earlier studies point to gaps in knowledge regarding formation and ongoing distribution of SNPs among higher organisms.

Massive sequence compendia from inbred murine strains furnish a powerful tool for investigating the “randomness” of SNP accumulation. In the present analysis, full genomic files from the Sanger Institute Mouse Genomes Project (http://www.sanger.ac.uk/resources/mouse/genomes/, last accessed August, 2015), including deep sequence and polymorphism data, were examined for 17 strains to investigate SNP formation bias. The murine strains (DBA, CBA, Balb/c, etc.) have each been backcrossed and/or inbred for well over 50 filial generations to reach allelic fixation with respect to ancestral polymorphism (Bailey 1978; Green 1981). Stringent parameters (depth of coverage, Phred quality score) were established to ensure that polymorphisms culled from the database were correctly delineated, and representative SNP cohorts were manually inspected to confirm authenticity. Our findings revealed a pronounced bias underlying SNP location in mice and we verified the same observations prospectively in human germ line DNA based on 1000 Genomes data (http://www.1000genomes.org, last accessed August, 2015) and acquired somatic mutations in human breast cancer (https://tcga-data.nci.nih.gov/tcga/dataAccess Matrix.htm, last accessed August, 2015). The results also establish a remarkable level of recent parallel evolution within the murine genome. Here we show regulatory patterns that underlie SNP formation, and provide a framework for investigating novel aspects of genomic diversification.

## Materials and Methods

### Acquisition of Data from Sanger Institute Compendium

SNP data from discrete regions (intronic, exonic, and intergenic) were queried and downloaded from the Sanger Mouse Genomes Project database (http://www.sanger.ac.uk/re sources/mouse/genomes/, last accessed August, 2015) for 17 highly inbred *Mus musculus* strains (Keane et al. 2011; Yalcin et al. 2011). Intronic and intergenic SNPs were obtained from chromosomes 1–3 after being shown to meet screening thresholds that included: 1) Sequencing depth of ≥30, and 2) Phred score of at least 60 for three categories (SNP quality, mapping quality, and consensus quality). Exonic SNPs were obtained from murine chromosomes 1–8 at the same level of sequencing depth and quality. In this manner, a very large region of chromosomal DNA (>500 million bp in each of 17 strains) was evaluated. Because 1) substantial variability in SNP patterns has not been suggested among different chromosomes; 2) previous studies in mouse, human, and other species have drawn valuable conclusions using comparable (or smaller) genomic samples (Zhang et al. 2005; Xue et al. 2009; Billings et al. 2010; Miller et al. 2012); and 3) practical aspects were found to limit a more extensive computational analysis, the chromosomal regions tested here were viewed as sufficient for purposes of this report. Polymorphism data were examined for every homozygous site for all 17 murine lines and with at least 4 strains (for noncoding DNA regions) or 2 strains (for exonic SNPs) bearing a minor allele in homozygous form. Positions of heterozygosity were also investigated. Because very few heterozygous alleles are anticipated among highly inbred (congenic) lines, well-validated heterozygous sites were provisionally interpreted as recent mutations (acquired during laboratory inbreeding [Bailey 1978; Green 1981; Silver 1995; Peters 2007; see also Considerations Regarding Murine Heterozygosity]). These data sets (termed “homosites” and “heterosites,” respectively) were distributed among six groups, according to type of SNP (A⇔G, C⇔T, A⇔C, etc.).

### Manual Inspection of Murine SNPs

As a further test of authenticity, a representative sampling of SNPs from each category (heterosite, homosite, exonic, intronic, and intergenic) and a random cohort of nucleotide positions without known polymorphism were evaluated using Interactive Genomics Viewer (IGV) software (http://www.broadinstitute.org/igv/, last accessed August, 2015). Primary data from the Sanger Mouse Genomes Project (http://www.sanger.ac.uk/re sources/mouse/genomes/, last accessed August, 2015) were downloaded and a 160–200 bp interval surrounding each SNP or random (non-SNP) position (selected by computer algorithm), and formally inspected for parameters associated with next generation sequencing (NGS) artifact including 1) diminished (local) Phred score, 2) low regional sequencing depth or map quality, 3) nearby short repeats (which suggest incorrect SNP alignment), 4) DNA motifs linked previously to sequence error (Dohm et al. 2008; Harismendy et al. 2009), 5) indels in the immediate vicinity, 6) obvious misalignment among multiple reads, 7) unexplained increase in reported coverage (i.e., “pile-up”; in which the number of sequences obtained over a particular DNA segment is markedly increased, indicating gene duplication and/or aberrant sequence alignment), and 8) evidence of greater than two haplotype blocks from the same region (suggestive of possible template contamination, as any gene in a particular murine strain should be represented by only two haplotypes). A screening algorithm for viewing the data was depicted in tabular form.

### Acquisition of Neighboring Nucleotide Context and Genomic Representation

Neighboring nucleotides that flank SNP positions were retrieved from the Ensembl genome browser (http://uswest.ensembl.org/Mus_musculus/Info/Index, last accessed August, 2015). DNA sequences 50 bp 5′ or 3′ to a position of interest were extracted from Ensembl so that reads surrounding each position could be compiled as output text files. The sequences were aligned at the time of acquisition and loaded into an Excel spreadsheet for analysis (see also Computer Simulation) with reverse complement SNPs combined (A⇔G with C⇔T, A⇔C with G⇔T). Base frequency at nucleotide positions relative to an SNP site (±50 bp) was monitored to obtain “bias (%)” (overall base representation across each respective region of the murine genome was subtracted from base representation observed experimentally for every relative position surrounding each SNP on murine chromosomes 1–8 [exonic] or 1–3 [intronic, intergenic]—larger coding DNA samples being necessary to record sufficient exonic variants). Phred score, depth cutoff, and so forth were held constant among all SNPs to maintain stringency. The analysis also was conducted for a comparable number of randomly chosen (nonpolymorphic) positions within murine chromosomes 1–4 (exonic) or 1–3 (intronic, intergenic), as further control to assess context-dependent SNP location. Standard deviation for base representation was measured by bootstrapping individual samples from the data set over a series of 2,000 repeats.

### Dinucleotide Quartet Analysis

For this study, dinucleotide quartets were defined as two base pairs upstream and two downstream of a polymorphic site or other nucleotide position being evaluated. The dinucleotide quartet frequency surrounding each SNP type was collected from 17 murine strains (DBA/2J, CBA/J, BALB/cJ, 129P2/OlaHsd, 129S1/SvImJ, 129S5SvEvBr, A/J, AKR/J, C3H/HeJ, C57BL/6NJ, CAST/EiJ, FVB/NJ, LP/J, NOD/ShiLtJ, NZO/HlLtJ, PWK/PhJ, WSB/EiJ) and normalized for expected values among all 256 possible quartets (5′-XX|XX-3′, where vertical line denotes SNP location) in areas of interest (exonic, intronic, and intergenic) as determined by a computer program that directly tallies occurrence of all possible quartets on murine chromosomes 1–3 using sequence data (.txt files) downloaded from Ensembl. The observed incidence for each quartet surrounding a particular SNP type was compared with a stochastic representation of SNP patterns (see Statistics). Quartets were considered “permissive” or “shielded” to polymorphism only if SNP representation was significantly different from expected; for example, for quartet context *E* surrounding an A⇔G SNP, *E* would be termed permissive for A⇔G variants only if observed association with single base replacement was statistically greater than the incidence at which both adenine and guanine are expected stochastically (only if *P* values for both nucleotides were significant).

### Acquisition and Analysis of Human SNP Data from 1000 Genomes

SNP data were queried and downloaded from 1000 Genomes (http://www.1000genomes.org, last accessed August, 2015) for 19 human genes of interest taken from a list extensively characterized by our laboratory for features, such as transition bias, genetic founder alleles, well-defined minor allelic frequency, intronic versus exonic SNP prevalence, haplotype block formation, synonymous versus nonsynonymous polymorphism, and conservation among multiple species (including horse, frog, zebrafish, opossum, shark, and chicken) (Fortini et al. 2000; Hill et al. 2014). Intronic DNA was studied to minimize evolutionary selection bias, and data were collated according to SNP type (A⇔G, C⇔T, A⇔C, etc.). Computer-based summation was used (as above) to measure incidence of each nucleotide context within human intronic DNA. Site-specific base frequencies (as well as “Bias (%)”) and dinucleotide quartet representation were calculated to determine over- or underrepresented contexts versus the incidence measured directly for each of 256 possible contexts across a greater than 300-million-bp region of the human introme.

### Analysis of Human Breast Cancer

SNP data from intronic regions were queried and downloaded from the TCGA database ([https://tcga-data.nci.nih.gov/tcga/dataAccessMatrix.htm, last accessed August, 2015] for somatic mutations specific to human breast carcinoma [i.e., differing from germ line]). Data were divided as above (A⇔G, C⇔T, A⇔C, etc.) and computer summation used to determine surrounding nucleotides for each somatic SNP. Site-specific base frequencies and dinucleotide quartet representation were calculated to determine over- and underrepresented contexts versus the incidence measured directly across greater than 300 million bases.

### Statistics

Because statistical analysis in this study involved comparisons between observed and expected SNP frequencies, as well as incidence of specific nucleotide contexts, *P* values were calculated by chi-square. We considered using Fisher’s exact test for analyses of the “hit” (SNP) and “no-hit” (no SNP) findings within 2 × 2 tables as magnitudes of these frequencies varied. However, chi-square using observed counts indicated that all assumptions for the test were satisfied. In particular, none of the four expected values in 2 × 2 contingency tables was less than 5 (Rosner 2011). We therefore concluded that chi-square was the appropriate test. Observed and expected SNP and context tallies were obtained (with frequencies calculated) and compared by 2 × 2 contingency tables with Yates’s correction for continuity (Yates 1934) to minimize Type I error. For tables of sequential tests (e.g., dinucleotide pairings in quartet representation; i.e., 256 sequential comparisons), the Bonferroni (Rice 1988) technique was used to further diminish Type I error. In other analyses, as a baseline for “CG” representation, triplet frequencies derived from murine codon usage (and equivalent counts of CG recognition by anticodons) were calculated and compared with a random distribution of dinucleotide frequency rates (based on computer summation of A, T, C, and G incidence from currently identified murine exonic DNA).

### Computer Simulation

**Fig. 1.—:**
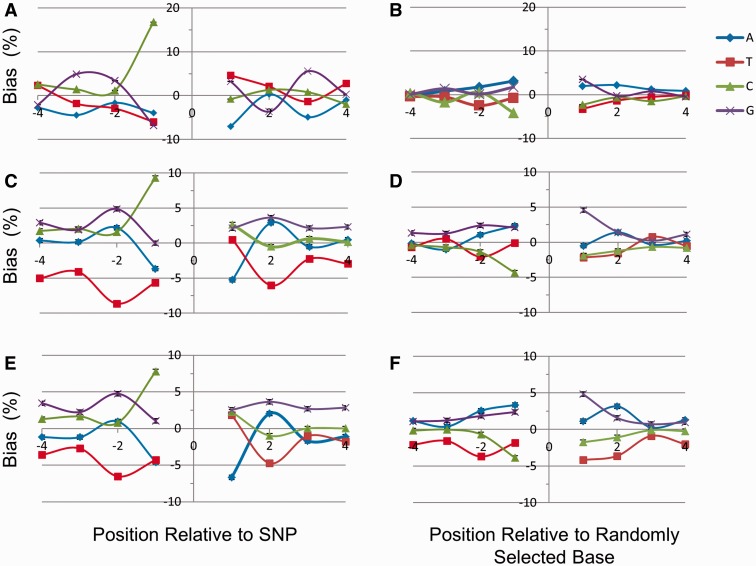
Base frequency bias of murine A⇔G SNPs. (*A*) Base location frequency bias (Bias %) immediately surrounding (±4 bp) 20,603 exonic A⇔G homozygous SNPs, compiled from coding regions of murine chromosomes 1–8. Bias % was calculated as described in Materials and Methods. (*B*) Genomic base frequency bias (±4 bp) relative to 20,000 randomly chosen (not SNP-associated) adenine (A) and guanine (G) nucleotides within murine exons (chromosomes 1–4) studied in a fashion otherwise identical to Panel (*A*). (*C*) Base location frequency bias immediately surrounding (±4 bp) 50,244 A⇔G SNPs compiled from intronic regions of murine chromosomes 1–3. (*D*) Genomic base frequency bias relative to 50,000 randomly chosen “A” or “G” sites within murine introns. (*E*) Base location frequency bias relative to 67,663 A⇔G SNPs compiled from intergenic regions on murine chromosomes 1–3. (*F*) Genomic base frequency bias relative to 50,000 randomly chosen “A” or “G” sites from intergenic regions of murine chromosome 2. In all cases, standard deviation (as judged by bootstrap analysis) was very low (on the order of ∼0.1–0.3%).

Computer programs (written in “java”) were designed to perform the following tasks. Specific sequences ±50 bp for a given SNP site were retrieved from the Ensembl genome browser (http://uswest.ensembl.org/Mus_musculus/ Info/Index, last accessed August, 2015). Randomly selected (nonpolymorphic) bases corresponding to a given SNP type were obtained as a control and aligned to the ±50-bp region surrounding these sites (i.e., for A⇔G SNPs, adenine or guanine sites were randomly selected). The program was run with Eclipse Integrated Development Environment (IDE) software (https://eclipse.org/downloads/packages/eclipse-ide-java-developers/marsr, last accessed August, 2015). Results were used as a further control for comparison to relative nucleotide frequencies associated with random SNP locations (see fig. 1). In addition, counts representing all dinucleotide quartets surrounding all base positions (A, C, G, or T) for downloaded genomic sequence (.txt files; e.g., exons, introns, and intergenic regions of murine chromosomes 1–3; >300 million bp of human intronic sequence) were established. These results served as an additional, independent control for evaluating quartet bias and SNP distribution.

## Results

### Context Biases Associated with Homozygous Murine and Hominid SNPs

To investigate positional bias contributing to SNP frequency and/or location, we studied genomic data from the Sanger compendium (Keane et al. 2011; Yalcin et al. 2011) stratified according to intergenic, intronic, or exonic regions of murine DNA. SNP data were filtered in our studies to include polymorphic sites for which multiple distinct murine strains in the repository encoded the minor allele in homozygous form. Sequence depth (≥30-fold coverage) and quality (Phred score) utilized by Sanger (Keane et al. 2011; Yalcin et al. 2011) were robust, and the convention of focusing on positions of redundant homozygous polymorphism allowed an additional level of stringency. For example, the probability of eight minor alleles being called incorrectly (e.g., due to sequence artifact) for a certain SNP identified as homozygous in 4 of 17 inbred murine strains by these criteria based on minimum sequence quality (i.e., Phred score) is less than 1 × 10^−^^22^. For any minor allele (i.e., the less common nucleotide in the population at a specific position), therefore, the likelihood of sequence artifact or miscall was remote.

Data were divided into six categories based on the type of SNP identified (A⇔G, A⇔C, A⇔T, etc.), and a computer algorithm established to retrieve and align surrounding nucleotides for each polymorphic site. In figure 1, exonic SNPs identified across chromosomes 1–8 in 17 murine strains were analyzed for position-specific bias. Because A⇔G transition SNPs (and reverse compliment C⇔T transitions on the opposite strand) are 1) present in higher numbers across the murine genome than other SNP categories, 2) of mechanistic interest, that is, enhanced in both pro- and eukaryotes (Collins and Jukes 1994; Wakeley 1994), and 3) were found to exhibit significantly conserved patterns in exonic, intronic, and intergenic regions, we focused the analysis on polymorphisms of this type. As described below, the same considerations also apply to other SNP categories.

A marked overrepresentation of cytosine (immediately 5′ to A⇔G SNP location) and underrepresentation of adenine (immediately 3′) were noted for exonic single base replacements (fig. 1*A*). This suggested a sequence bias in the immediate vicinity of A⇔G polymorphism. The same was observed prospectively for both intronic (fig. 1*C*) and intergenic (fig. 1*E*) murine SNPs (compared with randomly chosen controls; fig. 1*B*, *D*, and *F*). Bootstrapping indicated that standard deviations in all cases were small (0.1–0.3%; fig. 1). When data from 1000 Genomes for intronic SNPs among 19 randomly selected human genes were investigated, a similar pattern was observed (supplementary fig. S1*A*, Supplementary Material online). The same was true for somatic SNPs differing from germ line in human breast carcinoma (supplementary fig. S1*B*, Supplementary Material online).

We next investigated sets of “dinucleotide quartets,” or groups of four bases immediately 5′ or 3′ to each SNP site (X_1_X_2_**|**X_3_X_4_; where vertical line represents SNP location). Data are shown for A⇔G polymorphisms (table 1), but similar patterns were observed for other SNP categories (examples in supplementary tables S1–S3, Supplementary Material online). Each of 256 possible quartets was assembled and frequencies collated among homozygous SNPs for 17 murine strains in which 1) complete genomic sequence data were available and 2) multiple strains were homozygous for the minor allele at a specific position (see Materials and Methods). Statistical analysis was performed by comparing incidence of each quartet surrounding a particular SNP versus the observed (non-SNP associated) occurrence (as measured by computer summation of all possible quartets present on murine chromosomes 1–3 collated by exonic, intronic, and intergenic location). The results define a pronounced bias for SNP prevalence; ranking of quartets from among 256 possibilities for murine exonic A⇔G SNPs is shown in the far left column (table 1 and supplementary fig. S2, Supplementary Material online). Across murine exonic DNA (chromosomes 1–8; >20,000 SNPs), the 20 most frequent A⇔G SNP-associated quartets describe approximately 2,350 single nucleotide variants at preferential sites that would not have occurred on a random basis. For the 20 most shielding A⇔G contexts, approximately 1,840 SNPs expected at random were instead directed away from these specific motifs. Note that the quartets described here are not characteristic of stereotypic repetitive elements in human or murine DNA (LINE, SINE, ALU, B1 sequences, etc.). Moreover, the same SNP promoting or shielding contexts were observed in both exonic DNA (where ancient transposable or repetitive elements rarely occur) and the noncoding compartment, indicating that purifying selection for improved protein folding or function does not account for the contextual bias described here.

**Table 1:** Overrepresented (Permissive) 5′-XC|XX-3′ Quartets Surrounding A⇔G Transition SNPs

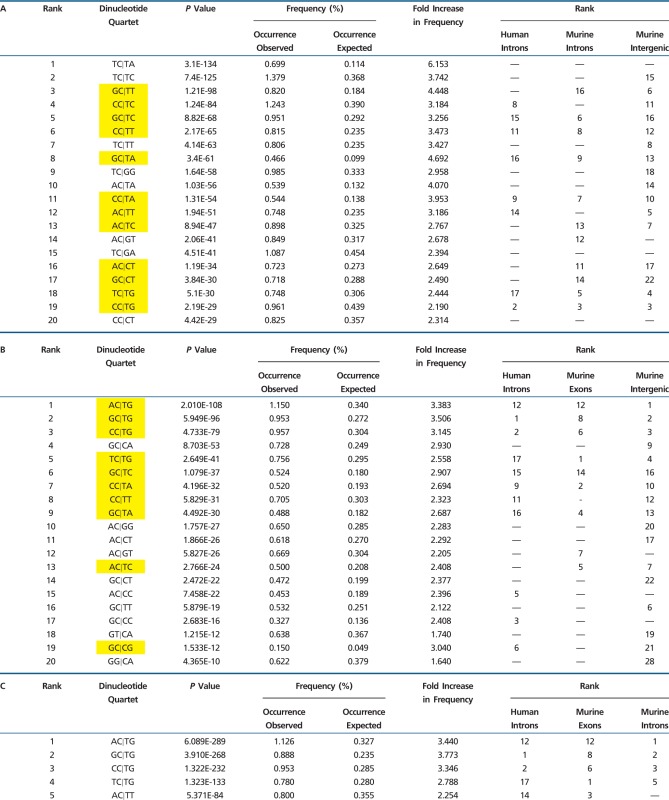
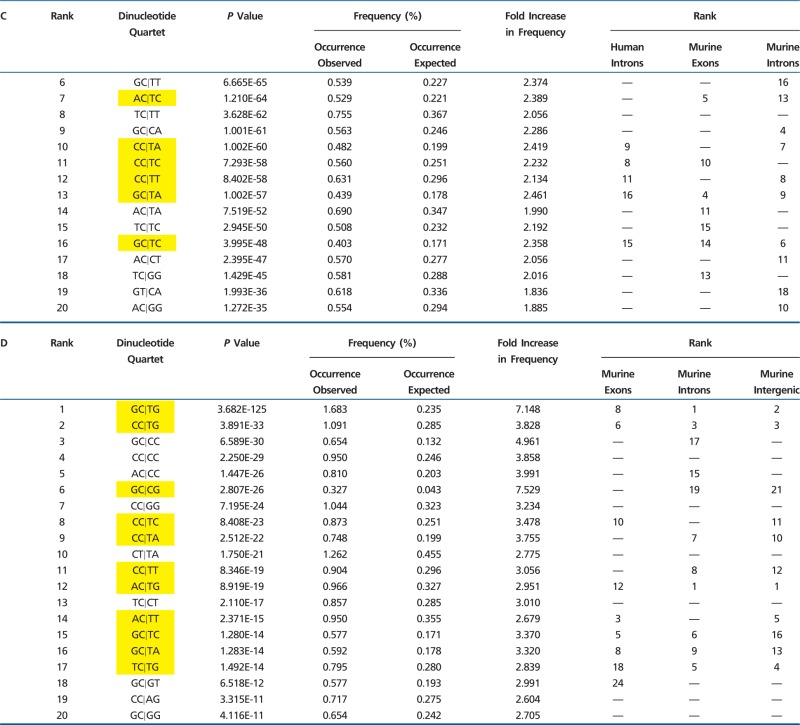

Note.—(A) Statistically overrepresented dinucleotide quartets surrounding 20,603 A⇔G coding (exonic) SNPs from murine chromosomes 1–8 (vertical line in each quartet indicates position of polymorphic base). Dinucleotide quartets were defined as two base pairs upstream and two downstream of a polymorphic site or other nucleotide position being evaluated. Rankings that strongly overlap between exonic murine SNPs and other murine and human SNP categories (quartets from among 256 possibilities significantly overrepresented in three of four murine and human DNA compartments) are indicated by yellow highlight; (B) same analysis for 50,244 intronic A⇔G SNPs from murine chromosomes 1–3; (C) findings for 67,663 intergenic A⇔G SNPs for murine chromosomes 1–3; (D) findings for 6,419 A⇔G SNPs from introns of 22 human genes. “—,” nonoverlapping in range shown. Note increased incidence of 5′ cytosine and 3′ thymine in quartet motifs predictive of SNP location.

The frequent observation of cytosine immediately 5′ to A⇔G polymorphism is likely attributable (at least in part) to DNA methylation on the complementary strand, followed by deamination (resulting in G:T mispairing). However, this cannot explain SNP-associated motifs in nontransition categories (e.g., A⇔C, C⇔G, etc.; supplementary tables S1 and S3, Supplementary Material online), nor does methylation account for overrepresentation of thymine (or dramatic underrepresentation of adenine) immediately 3′ to A⇔G base replacement (table 1). In addition, CG motifs were predictive not only of DNA transition but also A⇔C polymorphism (supplementary table S1, Supplementary Material online; note CG prominence immediately 5′ to A⇔C substitution [5′-CG|XX-3′]; a finding that cannot be attributed to cytosine methylation/deamination). Moreover, many of the 64 possible quartets with “C” preceding an A⇔G transition (5′-XC|XX-3′) showed no evidence of predisposition to SNP formation (table 2). Therefore, although a subset of transition mutations with 5′ cytosine are likely attributable to DNA methylation, this cannot account for either specificity or context of A⇔G SNPs identified here (preponderance of 5′-XC|TX-3′ and underrepresentation of 5′-XC|AX-3′), or the strong contextual patterns observed for other SNP categories.

**Table 2:** Nonpermissive 5′-XC|XX-3′ Quartets Surrounding A⇔G Transition SNPs

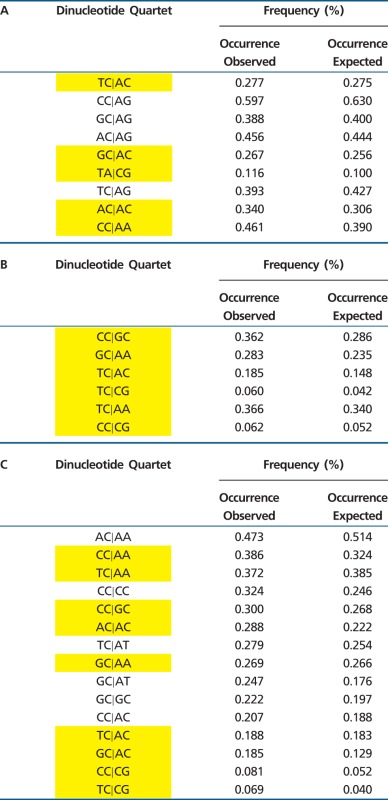

Note.—Nonpermissive 5′-XC|XX-3′ quartets in the setting of (A) 20,603 exonic (chromosomes 1–8), (B) 50,244 intronic (chromosomes 1–4), and (C) 67,663 intergenic (chromosomes 1–4) A⇔G homozygous SNPs. Vertical line in each quartet indicates position of polymorphic base. Yellow highlight indicates nonpermissiveness for SNP formation in at least two of the three regions shown in (A)–(C). Numerous contexts with cytosine immediately 5′ do not predict the location of an A⇔G SNP. All quartet *P* values are greater than 0.05 (Compare with table 1).

### Considerations Regarding Murine Heterozygosity

Heterozygosity among inbred murine strains was reported by Sanger when thresholds for sequencing accuracy were set at high stringency. Unlike the analysis described above which delineates homozygous SNP locations (the majority of which are typically attributed to alleles from genetic founders of murine lines and therefore comparatively ancient), authentic heterozygous positions are likely to reflect recent mutations on an otherwise highly inbred background (up to 290 filial inbreedings for certain strains tested here). For example, in a murine line that has undergone 50 filial crossings, 99.998% of ancestral heterozygous loci should be fixed; that is, only approximately 2 in 100,000 of the originally heterozygous positions should remain heterozygous (Bailey 1978; Green 1981; Silver 1995; Peters 2007). This calculation is conservative, as it does not account for finite genome size or the fact that physically linked SNPs are nonrandomly assorted (correction for either factor would substantially decrease likelihood of observing heterozygosity). Furthermore, because all classically inbred murine lines exceed filial generation 50 (F_50_), these mice are widely viewed as homozygous at every position in the genome (Peters 2007), barring a recent single nucleotide replacement. If one applies a conservative estimate of heterozygosity for ancestral (founder) alleles (e.g., 1 in 1,500 genomic positions), no more than 1 in 70 million locations among modern lines should remain heterozygous after 50 filial generations of inbreeding. Authentic heterozygosity is therefore likely to be quite recent.

A conceptual framework for estimating steady-state levels of population-based heterozygosity has been described previously (Charlesworth 2009; Lynch 2010). For example, the sex-averaged germline substitution rate among murine strains (μ) is approximately 30 × 10^−^^9^ SNPs per base pair per generation (Lynch 2010). An equilibrium level of heterozygosity (π_s_) can be calculated as 4N_e_μ (Charlesworth 2009; Lynch 2010), where N_e_ is the effective population size (a value of 2 in the setting of filial inbreeding). For a haploid genomic region of 500 Mb, therefore, approximately 120 (i.e., 4 × 2 × μ × 500,000,000 bp) steady-state heterozygous positions would be expected per generation on murine chromosomes 1–3 for each inbred strain, or 1,900 heterozygous positions among 16 murine lines analyzed here. (Murine strain AKR was omitted from the analysis based on an aberrantly high spontaneous mutation rate [Schlager and Dickie 1967].) This value represents a lower limit for the equilibrium level of SNPs, as the estimate does not account for somatic mutations early in development.

When we tested heterozygosity within murine chromosomes 1–3 (depth > 30; confidence [Phred] = 60) for 16 inbred strains, 18,558 heterozygous positions were observed. Note that Phred of 60 and depth of 30 represent very stringent benchmarks for SNP identification—the threshold is set to permit <<1 in a million miscalls from among approximately 18,500 heterosites. However, because approximately 120 heterosites should have been expected per murine line (i.e., ∼1,900 heterosites for 16 strains across ∼500 Mb of chromosomes 1–3), one must also consider the possibility that NGS artifact has led to a significant burden of erroneous SNPs.

### Studies to Minimize NGS Misalignment and Other Sequence Artifact

High-volume genomic sequencing is subject to miscalls, even when utilizing robust map quality, sequence depth, and Phred. With regard to the threshold for identifying authentic SNPs, we tested exonic, intronic, and intergenic DNA compartments in mice and human in a rigorous fashion to exclude sequencing error. The use of phred greater than 60 was incorporated to help assure absence of sequence artifact, which we confirmed by detailed inspection of representative SNPs and by utilizing regions with depth coverage greater than 30. Such criteria are exacting, but provide high levels of confidence in the data being evaluated. Supplementary tables S5–S7, Supplementary Material online, describe assessment of erroneous SNP assignment by manual inspection. Polymorphisms with low-quality score, surrounded by inconsistent consensus sequence data, artifactually high “coverage” (pile-up) due to homologous sequences elsewhere in the genome, or clearly duplicated reads, for example, can often be dispatched by direct visualization of a specific genomic interval. Other features of SNP environment—such as location within a short dinucleotide repeat or nearby indel—are sometimes more difficult to evaluate, as these regions are known to be genomically unstable, and represent common sites of true SNP formation (Pearson et al. 2005; Tian et al. 2008; Lopez Castel et al. 2010).

#### Manual Inspection of Representative Homosites

Supplementary table S5, Supplementary Material online, describes 150 intronic, exonic, and intergenic SNPs selected randomly from among homosites identified by this study. Because all homosites were 1) based on robust Phred score and sequencing depth, 2) required to exhibit the minor allele in multiple distinct strains, and 3) found to occur at roughly the expected incidence of genomic variation among murine lines (i.e., one SNP per every few thousand nucleotide positions), the prior likelihood of error was very low (estimated at <10^−^^22^ per SNP). This assertion is borne out by the absence of misalignment, duplicate reads, indels, short local repeats, and so forth in the majority of homozygous SNPs (supplementary table S5, Supplementary Material online). The pattern is similar to a randomly selected region of high-quality DNA sequence data (supplementary table S7, Supplementary Material online) and indicates that the substantial majority of homosites reported here is authentic.

#### Manual Inspection of Representative Heterosites

A significant number of heterosite positions are clearly artifactual and exhibit surrounding sequence misalignment, duplicate reads from multiple genomic regions, low quality, and so forth (supplementary table S6, Supplementary Material online). Nonetheless, a meaningful subset of representative heterosites (∼16%) fail to exhibit any evidence whatsoever of NGS artifact. These heterozygous SNPs exhibit high local mapping scores, depth of coverage, Phred, consistent surrounding sequence, and are without evidence of “pile-up,” local read duplication, homologues elsewhere in the genome, and so forth. The sampling analysis therefore suggests that from among 18,558 putative heterozygous positions, much smaller numbers (e.g., ∼16% or 3,000) are likely to represent the authentic sites of recent mutation. This agrees well with expected heterozygosity calculated above based on population accumulation and the known murine mutation rate (an estimated 1,900 heterozygous positions at steady state), particularly when one considers that de novo SNPs formed during early embryogenesis would further increase the total number of expected heterosites (i.e., by ∼2-fold) above the value shown here (Lynch 2010).

### Permissive and Nonpermissive SNP Contexts in the cDNA of Human Genes

**Fig. 2.—:**
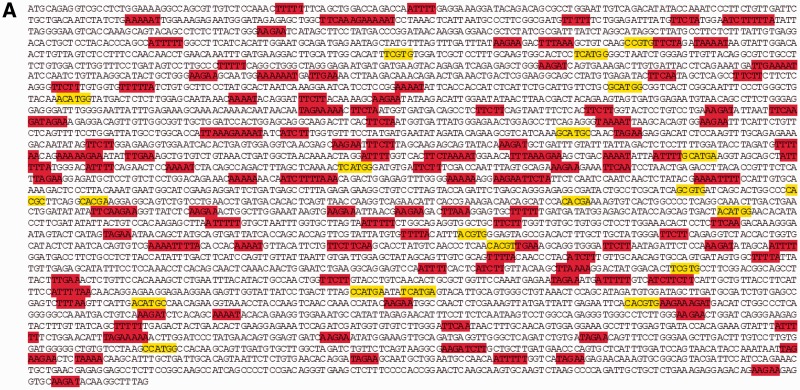

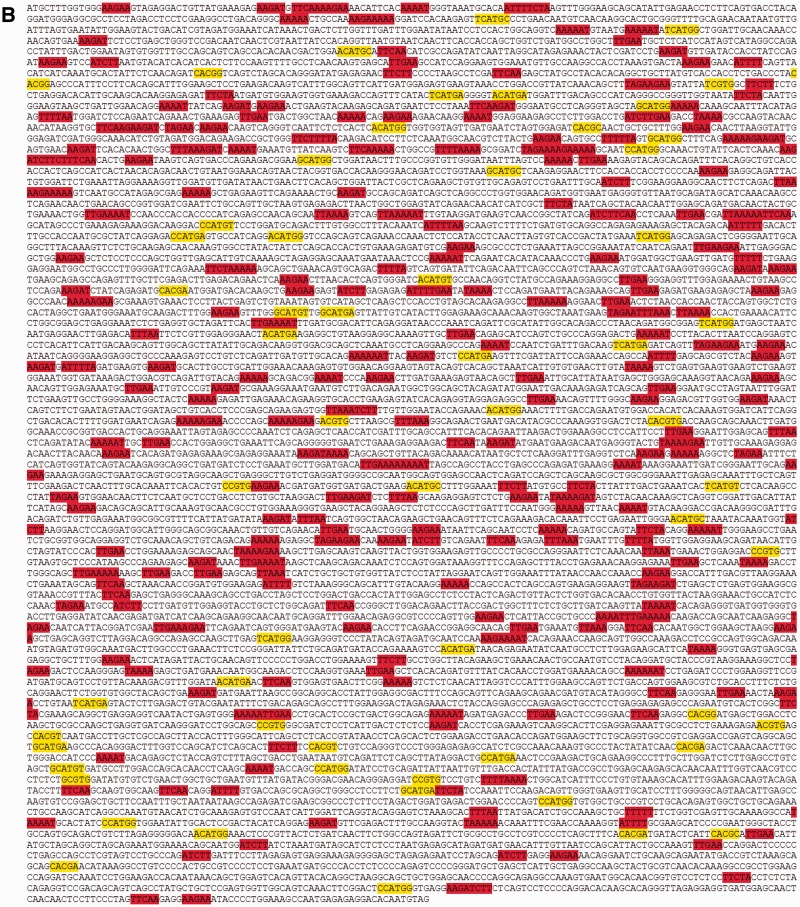
SNP-permissive and shielding quartets in human ORFs. (*A*) Most frequent SNP-permissive (yellow) and SNP-shielding (red) A⇔G quartet contexts within the cDNA of *CFTR*. (*B*) Same analysis for cDNA of dystrophin. A statistical (chi square 2 × 2 contingency table) analysis indicated overrepresentation of shielding dinucleotide quartets in both genes (*P* < 0.00001), and underrepresentation of quartets permissive for SNP formation (*P* < 0.00001).

We and others have suggested that random mutation accrual over billions of years could otherwise degrade the integrity of core metabolic genes, and that regulatory mechanisms may therefore exist to influence where (and possibly when) SNPs are most likely to occur in genomic DNA (Charlesworth B and Charlesworth D 1997; Loewe and Lamatsch 2008; Hill et al. 2014). One such mechanism is a transition bias that favors both synonymous and conservative exonic SNPs (Collins and Jukes 1994; Wakeley 1994; Freeland and Hurst 1998; Hill et al. 2014). To further investigate relevance of the present findings to exonic patterns of evolutionary SNP accumulation, we located the four greatest and four least permissive quartets for A⇔G polymorphism within cDNAs of *CFTR* and dystrophin, two genes of ancient vertebrate origin (fig. 2). We observed elevated representation of quartets that minimize SNP formation, and underrepresentation of motifs predisposed to augment the accrual of new SNPs. Either of these genes can be lethal when deleted from the mammalian genome, and therefore cannot be taken as representative of exonic DNA as a whole. However, because gene products such as these are likely to incur strong selective pressure, they provide a stringent (and nonneutral) test for SNP distribution bias. From this perspective, because the same contextual preferences shown here also apply across exonic, intronic, and intergenic DNA (fig. 1 and tables 1 and 2), the distributions cannot be ascribed to ongoing natural selection for optimizing or conserving amino acid sequence. Instead, we believe that constraints of this type offer a hint as to mechanisms that underlie SNP production in the murine genome (i.e., at the time of SNP formation, see below).

### Codon Usage, Exonic Dinucleotide Representation, and Relevance to SNP Formation Bias

The above analysis indicates that SNPs occur with greater frequency within the immediate vicinity of a CpG (i.e., 5′-CG|-3′), or within a CpG dinucleotide itself (5′-C|-3′). It is of interest that codon usage in mouse and human is underrepresented by CpG dinucleotides. For example, of the multiple nucleotide triplets available for serine, proline, threonine, and alanine (six for serine; four each for proline, threonine, and alanine), CG-containing codons are markedly underutilized. In mice, among six codons that designate serine, the triplet containing CG is preferred only 5.1% (*P* < 1.0 × 10^−^^30^; Materials and Methods). For proline, threonine, and alanine, the triplet containing CG is preferred at 10.3, 10.4, and 9.4%, respectively (*P* < 1.0 × 10^−^^30^ in all cases). Underrepresentation of CG dinucleotides within murine exons (supplementary fig. S3, Supplementary Material online) has been reported previously (International Human Genome Sequencing Consortium et al. 2001), connotes significance of the results shown in figure 2, and, based on findings presented here, would diminish exonic SNP formation and help preserve protein-coding DNA over the evolutionary timescale.

## Discussion

Our results demonstrate that nucleotide positions within murine and human DNA have distinct likelihoods of single base replacement that can be predicted in part from local sequence environment (e.g., see supplementary fig. S2, Supplementary Material online). A preference for transition substitutions (A⇔G and C⇔T), as well as transversion SNPs, was observed in the immediate vicinity of well-defined DNA quartets (fig. 1, supplementary figs. S1 and S2, Supplementary Material online, tables 1 and 2, and supplementary tables S1 and S3, Supplementary Material online). The findings are not compatible with a “neutral”-type DNA evolutionary model, as SNP accumulation genome wide is strongly nonrandom, as evidenced by local context, predisposition toward synonymous alterations (table 3), as well as a tendency to preserve exonic sequence (fig. 2 and supplementary fig. S3, Supplementary Material online). Frame of reference for these studies was based on expected incidence for random (i.e., stochastic) SNP accumulation.

**Table 3:** Comparison of Transition: Transversion and Nonsynonymous:Synonymous SNP Frequencies in Homozygous (Homosite) versus Heterozygous (Heterosite) Murine SNPs

SNP Category	Percent Transition	Percent Transversion	Nonsynonymous (NS) Coding SNPs	Synonymous (S) Coding SNPs	NS:S (*P* Value^a^)
Homozygous	77.7	22.3	708	1,245	1:1.76 (1.65E-14)
Heterozygous	66.3	33.7	272	425	1:1.56 (2.64E-11)

Note.—Murine homosites from chromosomes 1–3 with at least four lines exhibiting the minor allele are shown.

^a^*P* values represent observed SNP frequencies versus those that would be expected if SNPs formed stochastically (Materials and Methods).

The quartets described here are not attributable (at least in strict sense) to conventional evolutionary models in which SNP distribution is due to selective pressures that govern (along with drift, shift, hitchhiking, etc.) the tendency for allelic fixation. The same quartet preferences observed in coding DNA were also noted in noncoding (regulatory) DNA in mouse and human (yellow highlight, table 1), providing evidence that SNP-associated motifs are not a consequence of purifying selection, but are the result of a mechanism that governs their formation. This is particularly true with regard to the highly conserved sequences within nonprotein coding or regulatory DNA (ENCODE Project Consortium 2012; Gerstein et al. 2012), as there would be no reason to expect that the same quartets that provide an exonic (protein based) fitness advantage would also be advantageous for the intronic or intergenic DNA compartments. Our findings therefore support the existence of previously undescribed mechanism(s) by which single base replacements exhibit the same biased formation patterns in coding and noncoding, germline and somatic, and both murine and human DNA.

This study also investigated ostensibly more recent genetic polymorphism among 16 murine lines. Because these strains are heavily inbred (to over 50 filial matings), homozygosity at virtually every position would be expected, and the likelihood of a heterozygous site due to anything other than recent mutation is remote (Bailey 1978; Green 1981). Such strains are removed from many forms of selection that act during the evolutionary time scale; that is, most new single base replacements described above were generated in a “minimally selective” laboratory environment. We identified approximately 3,000 very recent SNPs across a 500-Mb region of DNA in multiple strains. The true extent of recent polymorphism must be significantly greater if one considers the high rate of fixation (drift; 1/2N_e_) for de novo heterosites (e.g., >90% of recent heterozygous positions are expected to become fixed after just 40 inbred generations [Bailey 1978; Green 1981]). This means a sizable majority of heterosites during 50–100 years of inbreeding should become fixed as homosites (typically retaining the original allele). Interestingly, when precise location for heterozygous SNPs was compared with sites with at least one (of 16) strain homozygous for the same minor allele, identity was substantial, in that 25–30% of all heterosites in one strain were also represented by a homosite in at least one other strain at exactly the same position. Moreover, although heterosites were rare, approximately 20% were represented in more than one strain as heterozygous. As the likelihood of a homosite miscall is negligible (<1 in 10^22^), such findings provide further support for the notion that significant numbers of heterosites have formed independently and in multiple strains (parallel evolution) and subsequently become “fixed”; that is, detected as homozygous SNPs of very high quality judged by Phred score, depth, mapping quality, and other criteria described above. For the frequent parallel variants described here (at least 20–25% of all recent point mutations), SNP formation is approximately 9 orders of magnitude greater than expected on a stochastic basis. Quantitative estimates of murine evolutionary rate have not previously considered parallel SNP formation or the positional constraints that appear to underlie a very large subset of newly accumulating SNPs.

Although it is expedient (and appealing) to simply attribute an unexpectedly large number of parallel SNPs to NGS artifact, we stringently tested this possibility and were unable to arrive at such a conclusion. Care was taken to exclude sequence artifact as a confounding variable. As noted above, the likelihood that a homozygous SNP in 4 of 17 murine strains represents sequence error is minimal (<1 in 10^22^ based on sequencing depth and high Phred quality) and manual SNP assessment indicates authenticity of the homosites identified here. In addition, DNA contexts found by our studies to predict SNP location have not been associated with sequencing error in the past, and motifs shown previously to increase rates of sequence artifact (e.g., SNPs preceded by “G” immediately 5′, poly-A tracts, etc. [Dohm et al. 2008; Harismendy et al. 2009]) were not identified as “permissive” (table 1 and supplementary tables S1–S3, Supplementary Material online). Manual analysis of local DNA environment did implicate NGS artifact as a primary source for most heterozygous calls, even at 30-fold coverage. Nonetheless, a meaningful subset of heterosites identified by Sanger appear authentic (supplementary table S6, Supplementary Material online). Also, as shown in table 3, enhancement of synonymous (vs. nonsynonymous) heterosite SNPs and a very strong transition bias (neither of which are associated with sequencing error) was observed in the heterosite population, further indicating authenticity. Heterozygous SNPs exhibited a synonymous:nonsynonymous ratio of approximately 1.6:1, which is very similar to the homosite value (1.76:1). Moreover, when we used established methods to estimate the equilibrium level of heterozygosity (π_s_) expected from germline mutation among 16 strains, we obtained a value of approximately 1,900 heterosites across 500 Mb (chromosomes 1–3), which is in reasonable agreement with a conservative estimate of approximately 3,000 authentic variants. Finally, even the most restrictive estimates indicate that meaningful numbers of heterozygous SNPs described by Sanger must exist. If all heterozygous positions are artifact, the present findings contradict known mutation rates in murine DNA, and debase a large number of past and ongoing genome-scale sequencing projects in multiple species, including human, that employ leading-edge data acquisition and analysis methods comparable to those used here. For the present interpretation of our findings to be discounted, therefore, one must assert that 1) parallel SNPs observed in our experiments are largely the result of NGS artifact (despite manual evaluation and other evidence to the contrary); 2) sequence artifact exhibits an inexplicable transition and synonymous bias, very similar to what occurs in living cells; 3) both heterozygous and homozygous calls are grossly in error (i.e., ∼25% of homozygosity is seriously tainted by NGS artifact); and 4) not only are the identified heterozygous SNPs incorrect but also the true heterozygous positions (of which 2,000–3,000 would be expected) are missing—and not detectable by the best available DNA sequencing technology.

As expected, well-validated heterozygous SNPs were rare, yet a high degree of parallel occurrence with homosites (25–30%) and other heterosites (20%) was observed after just a few decades of laboratory breeding. We believe that still higher levels of concordance would be obtained were additional time allowed for SNPs to accumulate, or if it were possible to directly measure the number of recent SNPs that have subsequently undergone fixation. In either case, the extent of strain propagation in the present studies essentially precludes ancestral haplotype as an explanation for observed patterns of heterozygosity, and instead points to a robust positional bias for recent mutation. The finding of context dependent, parallel, and recent SNP formation (many orders of magnitude beyond that predicted on stochastic basis) has not been considered in previous studies of murine genomic mutation rate (Ellison et al. 1996; Ananda et al. 2011), evolutionary “clocks” based on SNP genesis (Easteal et al. 1995; Hedges and Kumar 2003), ultravariable versus ultraconserved DNA otherwise ascribed to purifying selection (Ellison et al. 1996; Ahituv et al. 2007), or somatic mutational patterns in neoplasia (Song et al. 2013), but should be considered as part of future analyses in these areas.

We and others have characterized longevity of core metabolic genes in the face of a mutational “ratchet” (that over hundreds of millions of years would be capable of decimating eukaryotic exons), and suggested existence of adaptive mechanisms that regulate DNA mutation and serve to promote long-term genomic survival (Gabriel et al. 1993; Lynch 2010; Koonin 2012; Hill et al. 2014). Findings from the present study furnish new evidence in support of this hypothesis. We show that SNP distribution in congenic mice exhibits contextual bias that may divert single nucleotide variants away from protein-coding DNA (fig. 2 and supplementary fig. S3, Supplementary Material online). We also provide evidence that recently acquired (heterozygous) SNPs (produced in laboratory mice under minimal selective pressure) nonetheless exhibit a strong synonymous predisposition (table 3). In addition, our data point to modes of rapid DNA evolution restricted by specific sequences (e.g., CG dinucleotides) repletes in noncoding DNA (table 1 and supplementary table S1, Supplementary Material online). Contextual and other SNP preferences must, in part, reflect rates of nucleotide misincorporation, errors during proofreading, biased gene conversion, and/or failure to conduct mismatch-mediated repair with regard to certain DNA motifs. Findings presented here provide evidence that mutational fault tolerance has been adapted to spare eukaryotic reading frames. We also note that highly specialized sequence motifs favoring SNPs within promoters and other crucial regions of noncoding DNA (e.g., CpG islands) could serve to preferentially facilitate polymorphism and diversity in a manner that directs single base mutations to the regulatory compartment (ENCODE Project Consortium 2012; Gerstein et al. 2012), while shielding against uncontrolled mutation accrual within essential protein-coding elements (table 1 and supplementary table S1 and fig. S3, Supplementary Material online) (Hill et al. 2014). In either case, future studies of mouse genomic evolution should consider the role of contextual preference and parallel SNP formation shown here for highly inbred murine strains.

## Supplementary Material

Supplementary figures S1–S3 and tables S1–S7 are available at *Genome Biology and Evolution* online (http://www.gbe.oxfordjournals.org/).

Supplementary Data
