# Supplementary material for: SNP Formation Bias in the Murine Genome Provides Evidence for Parallel Evolution
Source: Genome Biol Evol. 2015 Aug 6;7(9):2506–19. doi: 10.1093/gbe/evv150 (PMC4607513; doi:10.1093/gbe/evv150)
Supplement: Supplementary Data [file supp_7_9_2506__index.html]

SNP Formation Bias in the Murine Genome Provides Evidence for Parallel Evolution — Supplementary Data 

# SNP Formation Bias in the Murine Genome Provides Evidence for Parallel Evolution

## Supplementary Data

files

- Supplementary Data - docx file
