# Supplementary material for: SNP Formation Bias in the Murine Genome Provides Evidence for Parallel Evolution
Source: Genome Biol Evol. 2015 Aug 6;7(9):2506–19. doi: 10.1093/gbe/evv150 (PMC4607513; doi:10.1093/gbe/evv150)
Supplement: Supplementary Data [file supp_evv150_SNP_Distribution_Supplementary_Figures_and_Tables.docx]

Supplemental Figure 1: Base frequency bias of human A⬄G SNPs.


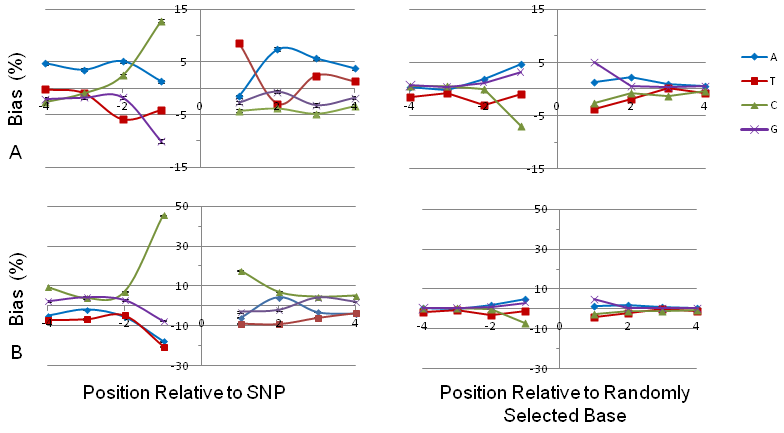


Supplemental Figure 1: (**A**) Left Panel: Base location frequency bias relative to intronic A⬄G human SNPs; 6419 A⬄G SNPs were analyzed from 19 human genes. Right Panel: Analysis of 7,000 randomly selected human non-coding ‘A’ or ‘G’ positions. (**B**) Left Panel: Base location frequency bias relative to 10,818 A⬄G human breast cancer somatic SNPs. Right Panel: Analysis of 7,000 randomly selected human intronic ‘A’ or ‘G’ positions (as in **A**; right Panel). (Note change in y-axis versus Figure 1). Bootstrap analysis indicated standard deviations < 0.6% for all data points shown above. Genes studied in **A** included CFTR, NF1, ABCA1, HTT, JAG1, MYH7, PTCH1, EXT1, ERCC4, SLC12A1, DMD, UBE3A, TBX5, CCND1, STK11, RPS6KA3, CAPN3, ERCC3, PAX6, selected at random from a list of well characterized human disease associated loci for which both exonic and intronic SNP data were available from 1000 Genomes.

Supplemental Figure 2: SNP quartet distribution for murine exonic A⬄G SNPs.


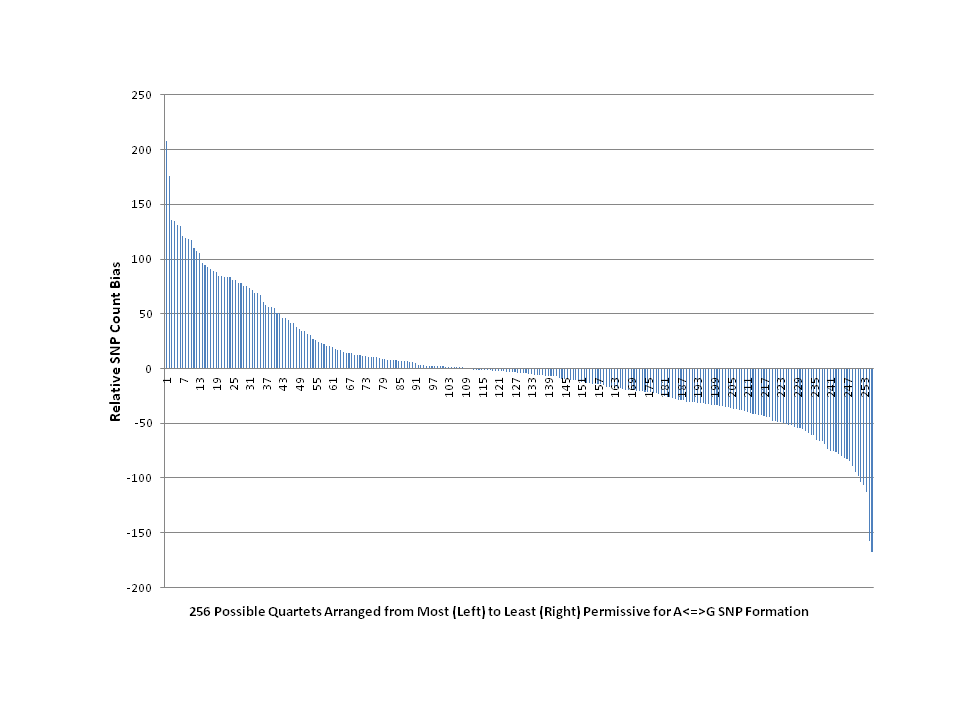


Supplemental Figure 2: Murine exonic A⬄G SNP bias for 256 possible dinucleotide quartets, ordered from most “permissive” (far left) to most “shielded” (far right). Refer to Supplemental Table 4 for a list of dinucleotide quartets shown above. ‘Relative SNP Count Bias’ was calculated by subtracting the number of expected SNPs within each context (based on the measured incidence of each quartet across murine chromosomes 1-4) from the number of SNPs observed for each context.

Supplemental Figure 3: Murine dinucleotide utilization in ORFs.


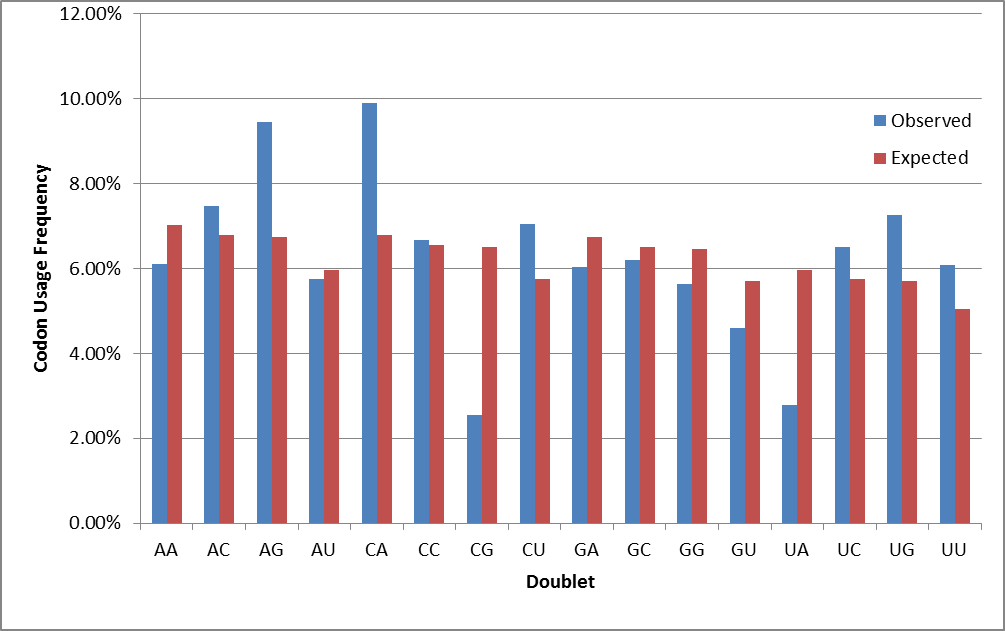


Supplemental Figure 3: Observed (actual, based on codon usage) and expected (based on random nucleotide utilization) dinucleotides within the exome of murine chromosomes 1-4. “Expected” values were calculated from measured A, C, T, and G frequencies. The diminished utilization of CG dinucletotides may reflect evolutionary selection against contexts strongly predisposed to SNP formation in mammalian exons (Table 1 and Supplemental Tables 1 and 3). In principle, diminished UA utilization could protect against premature termination codon formation (by avoiding new SNPs in proximity to UA nucleotide pairs).

Supplemental Table 1: Over-representation of certain 5’-CG|XX-3’ quartets surrounding A⬄C SNPs.

| *Rank*  A | Dinucleotide Quartet | p-value | Frequency (%) occurrence (observed) | Frequency (%) occurrence (expected) | Fold Increase in Frequency | Rank: Murine Introns | Rank: Murine Intergenic |
| --- | --- | --- | --- | --- | --- | --- | --- |
| 1 | CG\|AT | 1.139E-20 | 1.188 | 0.111 | 10.663 | 3 | 1 |
| 2 | CG\|AG | 1.634E-07 | 1.188 | 0.259 | 4.586 | 2 | 3 |
| 3 | CG\|GA | 8.722E-06 | 0.864 | 0.184 | 4.700 | - | - |
| 4 | CG\|GT | 3.036E-05 | 0.648 | 0.120 | 5.410 | 4 | 2 |
| 5 | TC\|GT | 3.716E-05 | 1.080 | 0.293 | 3.685 | - | - |
| 6 | CG\|GG | 1.146E-04 | 0.864 | 0.218 | 3.959 | - | - |

| Rank  B | Dinucleotide Quartet | p-value | Frequency (%) occurrence (observed) | Frequency (%) occurrence (expected) | Fold Increase in Frequency | Rank: Murine Exons | Rank: Murine Intergenic |
| --- | --- | --- | --- | --- | --- | --- | --- |
| 1 | CG\|AA | 7.868E-20 | 0.229 | 0.044 | 5.159 | - | 4 |
| 2 | CG\|AG | 3.189E-16 | 0.264 | 0.065 | 4.078 | 2 | 3 |
| 3 | CG\|AT | 3.575E-16 | 0.229 | 0.051 | 4.463 | 1 | 1 |
| 4 | CG\|GT | 4.092E-15 | 0.193 | 0.041 | 4.760 | 4 | 2 |
| 5 | AA\|CC | 1.299E-14 | 0.835 | 0.384 | 2.175 | - | 6 |
| 6 | CA\|GT | 1.347E-13 | 0.642 | 0.274 | 2.339 | - | - |
| 7 | CG\|TA | 1.035E-08 | 0.141 | 0.035 | 3.979 | - | 7 |
| 8 | AA\|CA | 1.069E-07 | 1.099 | 0.684 | 1.607 | - | 5 |
| 9 | AC\|CT | 9.384E-06 | 0.712 | 0.434 | 1.639 | - | - |

| Rank  C | Dinucleotide Quartet | p-value | Frequency (%) occurrence (observed) | Frequency (%) occurrence (expected) | Fold Increase in Frequency | Rank: Murine Exons | Rank: Murine Introns |
| --- | --- | --- | --- | --- | --- | --- | --- |
| 1 | CG\|AT | 2.26E-25 | 0.235 | 0.051 | 4.656 | 1 | 3 |
| 2 | CG\|GT | 2.64E-20 | 0.169 | 0.034 | 4.985 | 4 | 4 |
| 3 | CG\|AG | 1.43E-14 | 0.217 | 0.064 | 3.417 | 2 | 2 |
| 4 | CG\|AA | 1.68E-13 | 0.181 | 0.050 | 3.621 | - | 1 |
| 5 | AA\|CA | 7.38E-11 | 1.236 | 0.786 | 1.572 | - | 8 |
| 6 | AA\|CC | 3.22E-09 | 0.718 | 0.418 | 1.717 | - | - |
| 7 | CG\|TA | 8.36E-09 | 0.121 | 0.034 | 3.496 | 20 | 7 |

1. Statistically over-represented dinucleotide quartets surrounding 13,530 A⬄C coding (exonic) SNPs from murine chromosomes 1-8. Rankings that illustrate overlap between quartets for at least 2 of 3 murine genomic compartments are shown by yellow highlight, (**B**) same analysis for 11,378 intronic A⬄C SNPs from murine chromosomes 1-3, (**C**) findings for 16,585 intergenic A⬄C SNPs for murine chromosomes 1-3. Vertical line in each quartet indicates position of polymorphic base. ‘-‘ = non-overlapping in range shown

Supplemental Table 2: Non-permissive 5’-CG | XX-3’ quartets surrounding A⬄C SNPs.

| A | Dinucleotide Quartet | Frequency (%) occurrence (observed) | Frequency (%) occurrence (expected) |
| --- | --- | --- | --- |
|  | CG\|CA | 0.370 | 0.238 |
|  | CG\|CC | 0.251 | 0.191 |
|  | CG\|CT | 0.177 | 0.216 |

| B | Dinucleotide Quartet | Frequency (%) occurrence (observed) | Frequency (%) occurrence (expected) |
| --- | --- | --- | --- |
|  | CG\|CG | 0.009 | 0.012 |
|  | CG\|CC | 0.053 | 0.046 |
|  | CG\|CT | 0.053 | 0.063 |
|  | CG\|GG | 0.018 | 0.049 |
|  | CG\|GC | 0.070 | 0.040 |

| C | Dinucleotide Quartet | Frequency (%) occurrence (observed) | Frequency (%) occurrence (expected) |
| --- | --- | --- | --- |
|  | CG\|AC | 0.042 | 0.046 |
|  | CG\|CC | 0.042 | 0.044 |
|  | CG\|CT | 0.060 | 0.056 |
|  | CG\|CG | 0.012 | 0.013 |
|  | CG\|GC | 0.054 | 0.037 |
|  | CG\|CA | 0.090 | 0.058 |

Non-permissive 5’ – CG|XX – 3’ quartets in the setting of (**A**) 13,530 exonic, (**B**) 11,378 intronic, and (**C**) 16,585 intergenic A⬄C homozygous murine SNPs. Vertical line in each quartet indicates position of polymorphic base. Yellow indicates non-permissiveness for SNP formation in at least 2 of the 3 regions shown. All quartet p-values are >0.05 (compare to Supplemental Table 1).

Supplemental Table 3: Over-representation of specific 5’-XC|GX-3’ quartets surrounding C⬄G SNPs.

| A | Rank | Dinucleotide Quartet | p-value | Frequency (%) occurrence (observed) | Frequency (%) occurrence (expected) | Fold Increase in Frequency | Rank: Murine Introns | Rank: Murine Intergenic |
| --- | --- | --- | --- | --- | --- | --- | --- | --- |
|  | 1 | CC\|GG | 2.916E-141 | 2.077 | 0.318 | 6.535 | 1 | 8 |
|  | 2 | TC\|GG | 2.359E-133 | 1.625 | 0.216 | 7.540 | 3 | 9 |
|  | 3 | TC\|GT | 2.146E-96 | 1.053 | 0.128 | 8.213 | 2 | 15 |
|  | 4 | CC\|GA | 3.625E-70 | 1.384 | 0.263 | 5.270 | 4 | 3 |
|  | 5 | CC\|GT | 1.252E-61 | 0.903 | 0.138 | 6.529 | 9 | 10 |
|  | 6 | TC\|GA | 6.715E-51 | 1.068 | 0.212 | 5.035 | 12 | 6 |
|  | 7 | AC\|GT | 9.253E-48 | 0.813 | 0.139 | 5.833 | 10 | 1 |
|  | 8 | AC\|GG | 1.234E-36 | 0.873 | 0.190 | 4.605 | 5 | 18 |
|  | 9 | GC\|GG | 2.781E-35 | 1.023 | 0.252 | 4.059 | - | - |
|  | 10 | GC\|GT | 3.074E-29 | 0.707 | 0.156 | 4.541 | - | - |
|  | 11 | CC\|GC | 6.140E-27 | 0.948 | 0.264 | 3.596 | - | - |
|  | 12 | AC\|GA | 1.093E-25 | 0.813 | 0.212 | 3.825 | 6 | 11 |

| B | Rank | Dinucleotide Quartet | p-value | Frequency (%) occurrence (observed) | Frequency (%) occurrence (expected) | Fold Increase in Frequency | Rank: Murine Exons | Rank: Murine Intergenic |
| --- | --- | --- | --- | --- | --- | --- | --- | --- |
|  | 1 | CC\|GG | 4.374E-35 | 0.664 | 0.093 | 7.151 | 1 | 8 |
|  | 2 | TC\|GT | 5.372E-23 | 0.509 | 0.081 | 6.306 | 3 | 15 |
|  | 3 | TC\|GG | 1.810E-20 | 0.465 | 0.075 | 6.173 | 2 | 9 |
|  | 4 | CC\|GA | 4.998E-16 | 0.399 | 0.069 | 5.738 | 4 | 3 |
|  | 5 | AC\|GG | 2.471E-15 | 0.354 | 0.058 | 6.059 | 8 | 18 |
|  | 6 | AC\|GA | 3.578E-15 | 0.376 | 0.066 | 5.740 | 12 | 11 |
|  | 7 | CA\|AG | 1.357E-11 | 1.816 | 0.870 | 2.087 | - | 5 |
|  | 8 | CA\|AC | 6.962E-11 | 1.705 | 0.819 | 2.081 | - | 2 |
|  | 9 | CC\|GT | 1.021E-10 | 0.332 | 0.069 | 4.826 | 5 | 10 |
|  | 10 | AC\|GT | 5.091E-09 | 0.310 | 0.070 | 4.455 | 7 | 1 |
|  | 11 | CT\|TG | 1.008E-07 | 1.838 | 1.028 | 1.788 | - | 4 |
|  | 12 | TC\|GA | 1.792E-07 | 0.266 | 0.062 | 4.305 | 6 | 6 |

| C | Rank | Dinucleotide Quartet | p-value | Frequency (%) occurrence (observed) | Frequency (%) occurrence (expected) | Fold Increase in Frequency | Rank: Murine Exons | Rank: Murine Introns |
| --- | --- | --- | --- | --- | --- | --- | --- | --- |
|  | 1 | AC\|GT | 9.742E-19 | 0.350 | 0.063 | 5.575 | 7 | 10 |
|  | 2 | CA\|AC | 1.328E-15 | 1.829 | 0.880 | 2.079 | - | 8 |
|  | 3 | CC\|GA | 1.812E-15 | 0.350 | 0.072 | 4.839 | 4 | 4 |
|  | 4 | CT\|TG | 1.041E-14 | 1.845 | 0.911 | 2.026 | - | 11 |
|  | 5 | CA\|AG | 1.711E-13 | 1.797 | 0.908 | 1.980 | - | 7 |
|  | 6 | TC\|GA | 3.912E-13 | 0.286 | 0.058 | 4.938 | 6 | 12 |
|  | 7 | GT\|TG | 4.278E-13 | 1.750 | 0.885 | 1.976 | - | - |
|  | 8 | CC\|GG | 8.533E-12 | 0.366 | 0.094 | 3.893 | 1 | 1 |
|  | 9 | TC\|GG | 6.445E-11 | 0.302 | 0.072 | 4.169 | 2 | 3 |
|  | 10 | CC\|GT | 2.071E-10 | 0.270 | 0.062 | 4.337 | 5 | 9 |
|  | 11 | AC\|GA | 3.123E-10 | 0.286 | 0.069 | 4.127 | 12 | 6 |
|  | 12 | GA\|AG | 7.358E-09 | 1.495 | 0.827 | 1.808 | - | - |
|  | 13 | AA\|TG | 8.197E-07 | 1.098 | 0.607 | 1.809 | - | - |
|  | 14 | CT\|TC | 6.509E-06 | 1.352 | 0.829 | 1.632 | - | - |
|  | 15 | TC\|GT | 1.359E-05 | 0.223 | 0.070 | 3.190 | 3 | 2 |
|  | 16 | CA\|TC | 2.500E-05 | 0.843 | 0.471 | 1.790 | - | - |
|  | 17 | CA\|TA | 4.815E-05 | 0.779 | 0.434 | 1.794 | - | - |
|  | 18 | AC\|GG | 1.185E-04 | 0.191 | 0.062 | 3.077 | 8 | 5 |

(**A**) Statistically over-represented dinucleotide quartets surrounding 6,644 C⬄G coding (exonic) SNPs from murine chromosomes 1-8. Rankings that illustrate overlap between at least 2 of 3 murine genomic compartments are shown by yellow highlight, (**B**) same analysis for 4,517 intronic C⬄G SNPs from murine chromosomes 1-3, (**C**) findings for 6,288 intergenic C⬄G SNPs for murine chromosomes 1-3. ‘-‘ = non-overlapping in range shown.

Supplemental Table 4: Dinucleotide quartet reference for Supplemental Figure 2.

Supplemental Table 5A: Manual Inspection of Representative Exonic Homosites

| Chr | Location | Indel | Short local DNA repeats | Depth over 100* | Low quality reads or sequence misalignment | Possibly attributable to read pair elsewhere |
| --- | --- | --- | --- | --- | --- | --- |
| 1 | 9921025 |  |  |  |  | + |
| 1 | 13674185 |  |  |  |  |  |
| 1 | 15809136 |  |  |  |  | + |
| 1 | 34178970 |  |  |  |  |  |
| 1 | 34252720 |  |  |  |  |  |
| 1 | 44231090 |  |  |  |  |  |
| 1 | 66884873 | + |  |  |  |  |
| 1 | 89122749 |  |  |  |  |  |
| 1 | 92111340 |  |  |  | + |  |
| 1 | 109412145 |  |  |  |  |  |
| 1 | 120566033 |  |  |  |  |  |
| 1 | 130283739 | + |  |  |  |  |
| 1 | 137153681 |  |  |  |  |  |
| 1 | 137346641 |  |  |  |  |  |
| 1 | 137702192 |  |  |  |  |  |
| 2 | 51967483 |  |  |  |  |  |
| 2 | 119849832 |  |  |  |  |  |
| 2 | 120126667 |  |  |  |  |  |
| 2 | 120840375 | + |  |  |  |  |
| 2 | 122267362 |  |  |  |  |  |
| 2 | 129434643 |  |  |  |  |  |
| 2 | 143655881 |  |  |  |  |  |
| 2 | 151918817 |  |  |  |  |  |
| 2 | 155448172 |  |  |  |  |  |
| 2 | 164062810 |  |  |  |  |  |
| 3 | 35894832 |  |  |  |  |  |
| 3 | 36830019 |  |  |  |  |  |
| 3 | 79284737 |  |  |  |  |  |
| 3 | 103607538 |  |  |  |  |  |
| 3 | 126645918 |  |  |  |  |  |
| 3 | 126648232 |  |  |  |  |  |
| 3 | 134905172 |  |  |  |  |  |
| 3 | 151985324 |  |  |  |  |  |
| 3 | 159576790 |  |  |  |  |  |
| 4 | 9357081 |  |  |  |  |  |
| 4 | 11208232 |  |  |  |  |  |
| 4 | 46002995 |  |  |  |  |  |
| 4 | 69927851 |  |  |  |  |  |
| 4 | 99465392 |  |  |  |  |  |
| 4 | 118044796 |  |  |  |  |  |
| 5 | 67739262 |  |  |  |  |  |
| 5 | 75016032 |  |  |  |  |  |
| 5 | 107565980 |  | + |  |  |  |
| 5 | 116011461 |  |  |  |  |  |
| 5 | 124757030 |  |  |  |  |  |
| 5 | 131093844 |  |  |  |  |  |
| 5 | 137865225 |  |  |  |  |  |
| 6 | 83070787 |  |  |  |  |  |
| 6 | 83514181 |  |  |  |  |  |
| 6 | 120165410 |  |  |  |  |  |

Supplemental Table 5B: Manual Inspection of Representative Intronic Homosites

| Chr | Location | Indel | Short local DNA repeats | Depth over 100* | Low quality reads or sequence misalignment | Possibly attributable to read pair elsewhere |
| --- | --- | --- | --- | --- | --- | --- |
| 1 | 12906462 |  |  |  |  |  |
| 1 | 13179342 |  |  |  |  |  |
| 1 | 13217358 |  |  |  |  |  |
| 1 | 20802970 |  |  |  |  |  |
| 1 | 34271295 |  |  |  |  |  |
| 1 | 39376277 |  |  |  |  |  |
| 1 | 43217721 |  |  |  |  |  |
| 1 | 64745517 |  |  |  |  |  |
| 1 | 66284447 |  |  |  |  |  |
| 1 | 66907511 |  | + |  |  |  |
| 1 | 68576419 |  |  |  |  |  |
| 1 | 78701756 |  |  |  |  |  |
| 1 | 90138683 | + |  |  |  |  |
| 1 | 91370823 |  |  |  |  |  |
| 1 | 102317149 |  |  |  |  |  |
| 1 | 117816071 | + |  |  |  |  |
| 1 | 120532519 | + | + |  |  |  |
| 1 | 120756237 |  |  |  |  |  |
| 1 | 122154725 | + | + |  |  | + |
| 1 | 122163457 |  |  |  |  |  |
| 2 | 3084624 |  |  |  |  |  |
| 2 | 6044312 |  |  |  |  |  |
| 2 | 27205555 |  |  |  |  |  |
| 2 | 40758718 |  |  |  |  |  |
| 2 | 40949632 |  |  |  |  |  |
| 2 | 41957101 | + | + |  |  |  |
| 2 | 43721084 |  |  |  |  |  |
| 2 | 44895090 |  |  |  |  |  |
| 2 | 52161356 |  | + |  |  |  |
| 2 | 54703202 |  |  |  |  |  |
| 2 | 71116864 |  | + |  |  |  |
| 2 | 110633026 |  |  |  |  |  |
| 2 | 110651096 |  |  |  |  |  |
| 2 | 118506140 |  |  |  |  |  |
| 2 | 119505330 |  | + |  |  |  |
| 2 | 120007571 | + |  |  |  |  |
| 2 | 120227008 |  |  |  |  |  |
| 2 | 120703911 |  |  |  |  |  |
| 3 | 9523375 |  |  |  |  |  |
| 3 | 11824970 |  |  |  |  |  |
| 3 | 69350169 |  |  |  |  |  |
| 3 | 84714710 | + | + |  |  |  |
| 3 | 87482206 |  | + |  |  |  |
| 3 | 97745054 |  |  |  |  |  |
| 3 | 101928544 |  |  |  |  |  |
| 3 | 104668564 |  |  |  |  |  |
| 3 | 113769237 |  |  |  |  |  |
| 3 | 129302202 |  |  |  |  |  |
| 3 | 137886476 |  |  |  |  |  |
| 3 | 137891686 |  |  |  |  |  |

Supplemental Table 5C: Manual Inspection of Representative Intergenic Homosites

| Chr | Location | Indel | Short local DNA repeats | Depth over 100* | Low quality reads or sequence misalignment | Possibly attributable to read pair elsewhere |
| --- | --- | --- | --- | --- | --- | --- |
| 1 | 20791451 |  |  |  |  |  |
| 1 | 21089785 |  |  |  |  |  |
| 1 | 25892962 |  |  |  |  |  |
| 1 | 38831875 |  |  |  |  |  |
| 1 | 42611999 |  |  |  |  |  |
| 1 | 64233546 |  |  |  |  |  |
| 1 | 66170318 |  |  |  |  |  |
| 1 | 69320418 |  |  |  |  |  |
| 1 | 69330424 |  |  |  |  |  |
| 1 | 77198987 |  |  |  |  |  |
| 1 | 78739595 |  |  |  |  |  |
| 1 | 90720689 |  |  |  |  |  |
| 1 | 113954059 |  |  |  |  |  |
| 1 | 122213404 |  |  |  |  |  |
| 1 | 179207509 |  |  |  |  |  |
| 1 | 185458137 |  |  |  |  |  |
| 1 | 191820714 |  |  |  |  |  |
| 2 | 3557321 |  | + |  |  |  |
| 2 | 45727381 |  |  |  |  |  |
| 2 | 50578516 |  |  |  |  |  |
| 2 | 50873597 |  | + |  |  |  |
| 2 | 51338655 |  | + |  |  |  |
| 2 | 51564922 |  | + |  |  |  |
| 2 | 51834586 |  | + |  |  |  |
| 2 | 51876357 |  |  |  |  |  |
| 2 | 53202897 |  |  |  |  | + |
| 2 | 122871916 |  |  |  |  |  |
| 2 | 123141026 |  |  |  |  |  |
| 2 | 123460106 |  |  |  |  |  |
| 2 | 127524493 |  | + |  |  |  |
| 2 | 132949018 |  |  |  |  |  |
| 2 | 144881732 |  |  |  |  |  |
| 2 | 147806790 |  |  |  |  |  |
| 2 | 154113423 |  |  |  |  |  |
| 2 | 160320922 |  |  |  |  |  |
| 2 | 169989940 |  | + |  |  |  |
| 2 | 174441075 | + |  |  |  |  |
| 3 | 3001059 |  |  |  |  |  |
| 3 | 3001633 |  |  | + |  |  |
| 3 | 26137905 |  |  |  |  |  |
| 3 | 37770807 |  |  |  |  |  |
| 3 | 38085756 |  |  |  |  |  |
| 3 | 38691993 |  |  |  |  |  |
| 3 | 52673604 |  |  |  |  |  |
| 3 | 52679655 |  |  |  |  |  |
| 3 | 149154656 |  |  |  |  |  |
| 3 | 149203919 |  | + |  |  |  |
| 3 | 149603531 |  |  |  |  |  |
| 3 | 150401291 |  |  |  |  |  |
| 3 | 152736361 |  |  |  |  |  |

Supplemental Table 6A: Representative Exonic Heterosites

| Chr | Location | Indel | Short local DNA repeats | Depth over 100* | Low quality reads or sequence misalignment | Possibly attributable to read pair elsewhere |
| --- | --- | --- | --- | --- | --- | --- |
| 1 | 9535975 |  |  | + |  |  |
| 1 | 89952492 |  |  |  | + |  |
| 1 | 90135522 |  |  |  |  |  |
| 1 | 90144338 |  |  |  |  |  |
| 1 | 90173997 |  |  |  |  |  |
| 1 | 90174017 |  |  |  |  |  |
| 1 | 135251447 |  |  |  |  |  |
| 1 | 135251450 |  |  |  |  |  |
| 1 | 135253078 |  |  |  |  |  |
| 1 | 135253087 |  |  |  |  |  |
| 1 | 135253133 | + |  |  |  |  |
| 1 | 135255092 |  |  |  |  |  |
| 1 | 135260553 |  |  |  |  |  |
| 1 | 135262164 |  |  |  |  |  |
| 1 | 135262394 |  |  |  |  |  |
| 1 | 173510917 | + |  | + |  |  |
| 1 | 176030089 |  |  |  |  |  |
| 1 | 176030482 | + |  |  |  |  |
| 1 | 176030484 | + |  |  |  |  |
| 1 | 180026727 |  |  |  |  | + |
| 2 | 5882324 |  |  |  |  |  |
| 2 | 10499674 |  |  |  |  |  |
| 2 | 10499687 |  |  |  |  |  |
| 2 | 10499702 |  |  |  |  |  |
| 2 | 25486201 |  |  |  | + |  |
| 2 | 69587598 |  |  |  |  |  |
| 2 | 69587904 |  |  |  |  |  |
| 2 | 69587917 |  |  |  |  |  |
| 2 | 69588100 |  |  |  |  |  |
| 2 | 69588180 |  |  |  |  |  |
| 2 | 69588198 |  |  |  |  |  |
| 2 | 69588341 |  |  |  |  |  |
| 3 | 33700278 |  |  |  |  | + |
| 3 | 93007292 |  |  |  | + |  |
| 3 | 137289166 |  |  |  |  |  |
| 3 | 137810386 |  |  |  |  | + |
| 3 | 138114023 | + |  |  |  | + |
| 3 | 138114043 |  |  |  |  | + |
| 3 | 138114225 |  |  |  |  |  |
| 3 | 138114315 |  |  |  |  | + |
| 3 | 138114343 |  |  |  |  | + |
| 3 | 138114406 |  |  |  |  |  |
| 3 | 142274351 |  |  |  |  |  |
| 3 | 142274411 |  |  |  |  |  |
| 3 | 142287543 |  |  |  |  | + |
| 4 | 88248880 |  |  |  |  |  |
| 4 | 118566356 |  |  |  |  | + |

Supplemental Table 6B: Representative Intronic Heterosites

| Chr | Location | Indel | Short local DNA repeats | Depth over 100* | Low quality reads or sequence misalignment | Possibly attributable to read pair elsewhere |
| --- | --- | --- | --- | --- | --- | --- |
| 1 | 3231609 |  |  |  |  | + |
| 1 | 5083540 |  |  |  | + |  |
| 1 | 10322859 |  | + |  | + |  |
| 1 | 11840527 |  |  | + |  | + |
| 1 | 20226923 |  | + |  |  |  |
| 1 | 21030705 |  | + |  | + |  |
| 1 | 32500258 |  | + |  |  |  |
| 1 | 40070463 |  |  | + |  | + |
| 1 | 55627710 |  |  |  |  | + |
| 1 | 59047251 | + | + |  |  |  |
| 1 | 70436778 | + | + |  | + |  |
| 1 | 80554516 | + | + |  | + |  |
| 1 | 87515790 |  |  | + |  | + |
| 1 | 90000699 |  |  |  |  | + |
| 1 | 90114632 |  |  | + |  |  |
| 1 | 90173891 |  |  |  |  |  |
| 1 | 90204914 |  |  |  | + |  |
| 1 | 101824396 |  |  |  |  | + |
| 1 | 122166488 | + | + |  | + |  |
| 1 | 129867353 | + | + |  | + |  |
| 1 | 135251707 | + |  |  |  |  |
| 1 | 159106687 |  | + |  |  |  |
| 1 | 164358028 |  |  |  |  |  |
| 1 | 173512601 |  |  |  |  | + |
| 1 | 175843432 |  |  | + |  |  |
| 1 | 175901339 | + |  |  |  | + |
| 1 | 179925632 |  |  |  |  |  |
| 1 | 179926509 |  |  |  |  |  |
| 2 | 40477201 |  | + |  |  | + |
| 2 | 41035225 |  |  |  |  | + |
| 2 | 42106428 |  | + |  | + |  |
| 2 | 49566773 |  | + |  | + |  |
| 2 | 65571991 |  | + |  | + |  |
| 2 | 76004412 |  |  |  |  |  |
| 2 | 76004789 |  |  |  |  |  |
| 2 | 79860623 | + | + |  | + |  |
| 2 | 102505639 |  | + | + |  | + |
| 2 | 104496028 |  | + |  | + |  |
| 2 | 118508010 |  | + |  |  |  |
| 2 | 125670095 |  | + |  | + |  |
| 2 | 127335693 |  | + |  | + |  |
| 3 | 142272958 |  |  |  |  | + |
| 3 | 142274825 |  |  |  |  |  |
| 3 | 142277513 |  |  |  |  |  |
| 3 | 142280035 |  |  |  |  |  |
| 3 | 142280891 | + | + |  | + |  |
| 3 | 142287535 |  |  |  |  | + |
| 3 | 144408149 |  |  |  |  | + |
| 3 | 145089630 |  | + |  | + |  |
| 3 | 152607217 | + | + |  |  |  |

Supplemental Table 6C: Representative Intergenic Heterosites

| Chr | Location | Indel | Short local DNA repeats | Depth over 100* | Low quality reads or sequence misalignment | Possibly attributable to read pair elsewhere |
| --- | --- | --- | --- | --- | --- | --- |
| 1 | 173443118 |  |  |  |  |  |
| 1 | 173497778 |  |  |  | + |  |
| 1 | 175879718 |  |  | + |  | + |
| 1 | 179842389 |  | + |  |  |  |
| 1 | 179842537 |  |  |  |  | + |
| 1 | 179887130 |  |  |  |  |  |
| 1 | 179887245 |  |  |  |  |  |
| 1 | 179902268 |  |  |  |  | + |
| 1 | 179924578 |  |  |  |  |  |
| 1 | 179924786 |  |  |  |  |  |
| 1 | 180080828 |  |  |  |  | + |
| 1 | 185453051 |  |  |  | + | + |
| 1 | 189282672 |  | + |  | + |  |
| 2 | 34389572 |  | + |  | + |  |
| 2 | 37594048 |  | + |  | + |  |
| 2 | 42515858 | + | + |  | + |  |
| 2 | 47090442 |  | + |  | + |  |
| 2 | 53568237 |  |  |  |  | + |
| 2 | 96732855 |  | + |  |  |  |
| 2 | 98632642 |  | + |  | + |  |
| 2 | 104496016 |  | + |  | + |  |
| 2 | 106549305 |  | + |  | + | + |
| 2 | 115648681 |  | + |  | + |  |
| 2 | 119464567 |  | + |  | + |  |
| 2 | 129660344 |  | + |  | + |  |
| 2 | 138772713 |  | + |  | + | + |
| 2 | 150999817 |  |  |  |  | + |
| 2 | 157900785 |  |  |  | + | + |
| 2 | 167967420 | + | + |  | + |  |
| 2 | 170030114 |  | + |  | + |  |
| 3 | 3052178 |  |  |  |  | + |
| 3 | 5910264 |  |  |  |  | + |
| 3 | 15329871 |  |  |  |  |  |
| 3 | 15332818 |  |  | + |  |  |
| 3 | 34057688 |  |  |  | + | + |
| 3 | 54138831 |  |  |  |  | + |
| 3 | 70170729 |  | + |  | + |  |
| 3 | 73474903 | + | + |  |  |  |
| 3 | 79566352 |  |  | + |  | + |
| 3 | 93567633 |  | + |  |  |  |
| 3 | 99021127 |  |  |  |  |  |
| 3 | 100354599 |  | + |  |  |  |
| 3 | 115366061 | + | + |  | + |  |
| 3 | 134253681 |  | + |  |  |  |
| 3 | 142278260 |  |  |  |  | + |
| 3 | 142696966 |  | + |  | + | + |
| 3 | 147557529 |  | + |  | + | + |
| 3 | 148782341 | + | + |  | + |  |
| 3 | 149642057 | + | + |  | + |  |
| 3 | 156172427 |  |  |  |  | + |

Supplemental Table 7: Representative Random Sites

| Chr | Location | Indel | Short local DNA repeats | Depth over 100* | Low quality reads or sequence misalignment | Possibly attributable to read pair elsewhere |
| --- | --- | --- | --- | --- | --- | --- |
| 1 | 14059518 |  |  |  |  |  |
| 1 | 16082562 |  |  |  |  |  |
| 1 | 24971269 |  |  |  |  |  |
| 1 | 27174060 | + |  |  |  |  |
| 1 | 28269747 |  |  |  |  |  |
| 1 | 31170136 | + |  |  | + |  |
| 1 | 43628573 |  |  |  |  |  |
| 1 | 69382824 |  | + |  |  |  |
| 1 | 87897453 |  |  |  |  |  |
| 1 | 102451441 |  |  |  |  |  |
| 1 | 105369585 |  |  |  |  |  |
| 1 | 112031529 |  |  |  |  |  |
| 1 | 114820480 |  |  |  |  |  |
| 1 | 122931128 |  | + |  |  |  |
| 1 | 133767479 |  |  |  |  |  |
| 1 | 135002952 |  |  |  |  |  |
| 2 | 9674167 |  |  |  |  |  |
| 2 | 18313975 |  |  |  |  |  |
| 2 | 33396713 |  |  |  |  |  |
| 2 | 35748371 |  |  |  |  |  |
| 2 | 42112468 |  |  |  |  | + |
| 2 | 76560240 |  |  |  |  |  |
| 2 | 89691569 |  |  |  |  |  |
| 2 | 101152169 |  |  |  |  |  |
| 2 | 103902341 | + |  |  |  |  |
| 2 | 115917965 |  |  |  |  |  |
| 2 | 129993907 |  |  |  |  |  |
| 2 | 144626361 |  |  |  |  |  |
| 2 | 145656086 |  |  |  |  |  |
| 3 | 9936548 | + | + |  |  |  |
| 3 | 14934114 |  |  |  |  |  |
| 3 | 26136024 |  |  |  |  |  |
| 3 | 31654325 | + |  |  |  |  |
| 3 | 32075071 |  |  |  |  |  |
| 3 | 34872444 |  |  |  |  |  |
| 3 | 42248345 |  |  |  |  |  |
| 3 | 45909329 |  |  |  |  |  |
| 3 | 50238577 |  |  |  |  |  |

Legend for Supplemental Tables 5-7:

SNPs were selected randomly from the categories delineated above (homosites, heterosites, exonic, intronic, intergenic) – or as random non-polymorphic positions across murine chromosomes 1-3. These were assessed using Integrative Genomics Viewer (IGV) software (Methods, *Manual Inspection of Murine SNPs*). An interval of 150-190bp was analyzed surrounding each SNP, and scored by two investigators for the parameters shown. Presence of indels was determined by noting an insertion or deletion in a majority of reads that included the SNP under study. “Low quality reads” or “sequence misalignments” were determined by noting whether a SNP was surrounded by multiple non-aligned bases in comparison to the consensus sequence (homosites and heterosites) and whether one SNP allele among the many reads was solely associated with multiple non-aligned bases (heterosites). SNPs (and random positions) conservatively defined as 1) artifact based on manual inspection or 2) possibly artifact if found in a short local repeat (known to represent regions of genomic instability and SNP formation, and less likely to represent sequencing error in the absence of indel) are shown without shading. SNPs (and random positions) designated as likely authentic (absence of any indication of a ‘miscall’) are indicated by blue shading.

*Suggests artifactually high coverage due to homologous sequences elsewhere in the genome.
